# Application of Comb-Type Fluorocarbon Polymer Surfactants as Dispersants in Insecticidal and Fungicidal Suspension Concentrates

**DOI:** 10.3390/molecules30204034

**Published:** 2025-10-10

**Authors:** Jingjing Zhang, Lingyun Song, Ruiying Ma, Junjie Kou, Yuxiu Liu, Qingmin Wang

**Affiliations:** 1 College of Basic Science, Tianjin Agricultural University, Tianjin 300392, China; 2 State Key Laboratory of Elemento-Organic Chemistry, Frontiers Science Center for New Organic Matter, College of Chemistry, Nankai University, Tianjin 300071, China; song13864482671@163.com (L.S.); 15641108088@163.com (R.M.); kjj@nankai.edu.cn (J.K.)

**Keywords:** polymer surfactant, comb-type, application, suspension concentrate

## Abstract

To investigate the application of novel comb-type fluorinated polymeric surfactants in suspension concentrates (SCs), we used the optimized surfactants (Comb-S 1, Comb-S 2, and Comb-S 3) as dispersants, with commercial dispersants as controls, to prepare 15% indoxacarb SC and 22% iprodione SC, respectively. The physicochemical properties (including suspension rate, particle size, and stability) and indoor biological activity of the prepared SC were determined. Comprehensive data indicated that for 15% indoxacarb SC, the novel comb-type fluorinated polymeric surfactants exhibited good compatibility with commercial dispersants. Whether used in combination or alone, SC demonstrates excellent dispersibility, stability, and application efficacy. For 22% iprodione SC, Comb-S 2 demonstrated good compatibility with commercial dispersants. Furthermore, the fungicidal activity test against *Botrytis cinerea* showed that Comb-S 2 could not only replace commercial dispersants, but the 22% iprodione SC formulated with Comb-S 2 exhibited more outstanding fungicidal activity.

## 1. Introduction

Suspension concentrate (SC), a water-based pesticide formulation, exhibits advantages such as high efficacy, long-lasting effect, good water solubility, small particle size, low cost, and excellent environmental compatibility [1,2]. However, as a thermodynamically unstable polydisperse system, water separation, layer separation, sedimentation, flocculation [3,4], coalescence, and Ostwald ripening [5,6,7] tend to occur during storage.

Fluorosurfactants are obtained by fully or partially replacing hydrogen atoms in the hydrophobic chains of hydrocarbon surfactants [8,9,10,11,12,13]. They can be used as wetting agents or dispersants in pesticide formulation. Compared to hydrocarbon surfactants, fluorosurfactants exhibit stronger hydrophobicity, superior surface activity, and higher surface activity at lower concentrations [14].

Compared with ionic fluorosurfactants, non-ionic fluorosurfactants have higher emulsifying capacity, better wettability, stability, and acid–base resistance. Additionally, they exhibit good compatibility with ionic and amphoteric surfactants, allowing for their use in compound formulations. Non-ionic fluorosurfactants have been widely applied in various fields, including pesticides, pharmaceuticals, and fire protection [15,16]. Polyethylene glycol is a non-toxic hydrophilic compound with excellent biocompatibility. It has been widely used in the synthesis of non-ionic fluorosurfactants [17,18,19,20,21].

Eastoe et al. [17] used triethylene glycol monomethyl ether, fluoroalcohol, and other substances as raw materials. They prepared four types of non-ionic fluorosurfactants with the structure X-(CF_2_)_m_CH_2_O(C_2_H_4_O)_3_CH_3_ (where X = F or H, m = 4 or 6) via a two-step substitution reaction. Mecozzi et al. [18] used sulfonylated polyethylene glycol monomethyl ether and perfluoroalkyl iodide as raw materials. By adjusting the molecular weight of polyethylene glycol monomethyl ether and the lengths of hydrocarbon chains and fluorocarbon chains, they designed and synthesized Y-shaped polymers with two fluorine-containing hydrophobic chains. Haag et al. [19] investigated surfactants composed of triblock copolymers, which are based on perfluoropolyether and polyethylene glycol blocks with droplet-based microfluidics. Wang et al. [21] designed and synthesized 10 new types of non-ionic fluorosurfactants with different hydrophilic and hydrophobic chain lengths, using polyethylene glycols and fluoroalcohols as raw materials. They systematically investigated the static and dynamic surface tensions of these surfactants and studied their structure-activity relationships.

Compared with conventional surfactants, polymeric surfactants exhibit advantages of large steric repulsion, a high number of anchoring groups per molecule, and strong surface adsorption [22,23]. Owing to their large molecular weight and diverse chain structures, polymeric surfactants can generate a steric barrier through adsorption. This phenomenon may be associated with unfavorable chain entanglement and entropic effects, which contribute to enhanced stability.

Comb-type polymeric surfactants are characterized by easily tunable structures and excellent dispersibility. In our previous work, we synthesized novel comb-type fluorinated polymeric surfactants (named as poly(PEGMEMA*_m_*-FMA*_n_*)) via free radical polymerization. For these surfactants, poly(ethylene glycol) monomethyl ether methacrylate (PEGMEMA) with different molecular weights was used as the hydrophilic segments, while fluoroalkyl methacrylate (FMA) with varying fluorocarbon chain lengths [(CF_2_)*_n_*, *n* ≤ 6] served as the hydrophobic segments. Through previous research, we screened out three polymeric surfactants (Comb-S 1, Comb-S 2, and Comb-S 3) with excellent overall performance (Table 1) [24].

Surfactants can act as dispersants, adsorbing onto the surface of technical material particles. They work by effectively wetting the particles. They penetrate and displace the air trapped between them. This action reduces the interfacial tension between the liquid–liquid (or solid–liquid) phases, which minimizes particle aggregation within the dispersion system and ultimately enhances dispersibility [25]. Therefore, we selected 15% indoxacarb suspension concentrate (SC) and 22% iprodione SC as models, and investigated the application of commercial dispersants (SP-27001, SP-SC29, YUS-FS3000) as well as the selected surfactants (Comb-S 1, Comb-S 2, Comb-S 3) in 15% indoxacarb SC and 22% iprodione SC, respectively. Through tests on the performance indicators and bioactivity of the obtained indoxacarb SC and iprodione SC, we discussed in detail the application of comb-type fluorinated polymeric surfactants as pesticide dispersants.

## 2. Results and Discussion

### 2.1. Content and Suspensibility

#### 2.1.1. Content and Suspensibility of 15% Indoxacarb SC

**Figure 1:**
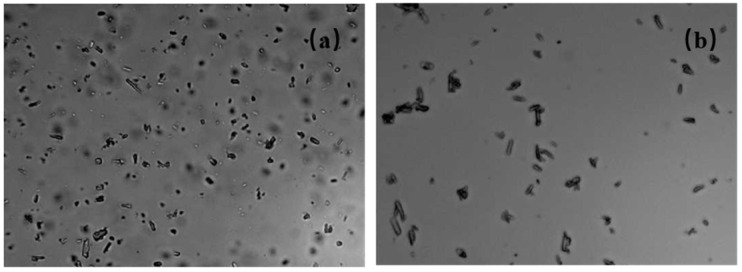
Morphology of 15% indoxacarb SC particles under an optical microscope (400× magnification). (**a**) 15% indoxacarb SC 2# (0 °C); (**b**) 15% indoxacarb SC 2# (54 °C).

As in Table 2, commercial dispersants, Comb-S 1, Comb-S 2, and Comb-S 3 were used to prepare 15% indoxacarb SC, namely 15% indoxacarb SC 1#, 2#, 3#, and 4#. The suspension rate of the SC decreases under both thermal storage (54 °C) and freezing conditions (−18 °C). Under 54 °C, particles exhibit vigorous motion, which may lead to collisions and subsequent aggregation into larger entities. Moreover, the system may undergo Ostwald ripening. Large particles in the formulation grow at the expense of small ones, as shown in Figure 1. In contrast, at −18°C (below the freezing point of water), ice formation excludes and compresses suspended pesticide particles. These particles are confined in a narrow space and forced to be at extremely close distances, thus aggregation is more likely. Overall, high-temperature storage presents a more significant challenge to formulation stability compared to low-temperature conditions [26].

The combinations of Comb-S 2/SP-SC29 and Comb-S 3/SP-SC29, as well as the use of Comb-S 1 alone, all exhibit excellent dispersibility and stability. Our surfactants are also highly compatible with other types. Notably, Comb-S 1 alone can effectively replace the need for traditional dispersants in compounds for 15% indoxacarb SC.

#### 2.1.2. Content and Suspensibility of 22% Iprodione SC

As shown in Table 3, commercial dispersants (SP-SC29, SP-27001, and YUS-FS3000) and Comb-S 2 were used to prepare 22% iprodione SC, namely 22% iprodione SC 1#, 2#, 3#, 4#, and 5#. After 22% iprodione SC was stored for 14 days under three conditions (0 °C, −18 °C, and 54 °C), their active ingredient contents ranged from 22.60% to 23.62%. Moreover, the variation range of the active ingredient contents was small under the three conditions. No decomposition or other adverse changes in the active ingredient occurred under these storage conditions.

For samples 1# and 3#, their suspension rates under −18 °C conditions were 56.48% and 65.36%, respectively. These suspension rates are relatively low, meaning that 22% iprodione SC 1# and 3# are not suitable for long-term storage under −18 °C. Further analysis of their formulations revealed that SP-27001 is not suitable for the freezing storage (−18 °C) of 22% iprodione SC.

The suspension rates of 22% iprodione SC 2#, 4#, and 5# were all above 90%. This indicates that whether using the commercial dispersants (SP-SC29 and YUS-FS3000) or Comb-S 2 meets the standards for commercial application, exhibiting good dispersing effects on 22% iprodione SC.

### 2.2. Particle Size and Morphology Characterization

**Figure 2:**
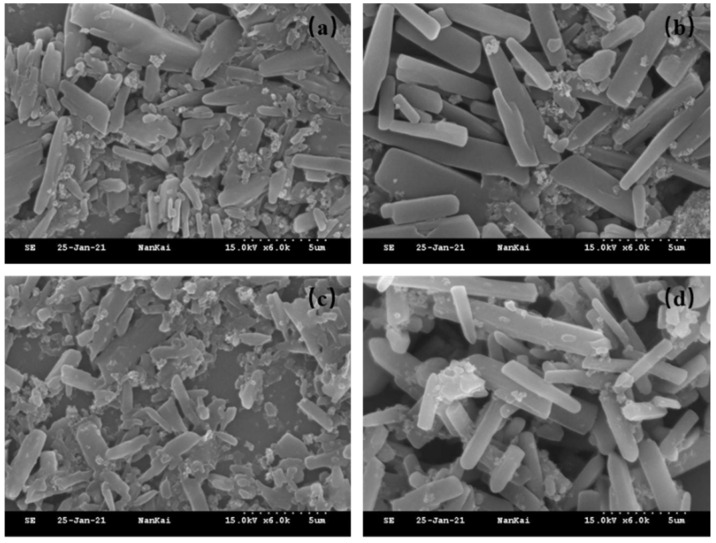
Morphology of 15% indoxacarb SC particles under a scanning electron microscope (SEM). (**a**) 15% Indoxacarb SC sample 1# (0 °C); (**b**) 15% Indoxacarb SC sample 1# (54 °C); (**c**) 15% Indoxacarb SC sample 2# (0 °C); (**d**) 15% Indoxacarb SC sample 2# (54 °C).

As shown in Table 2, the particle sizes of 15% indoxacarb SC (1#, 2#, 3#, 4#) under 0 °C were 4.964, 3.520, 4.208, and 4.550 μm, respectively. Among these storage temperatures, 15% indoxacarb SC 2# had the smallest particle size. After 54 °C storage (Figure 2), the SC particle size showed a significant increase due to Ostwald ripening [27]. Combined with the data in Table 2, 15% indoxacarb SC 3# exhibited comparable particle size and suspension rate to 15% indoxacarb SC 1# under all three storage conditions. This indicates that dispersants Comb-S 1, Comb-S 2, and Comb-S 3 exhibit good dispersibility for 15% indoxacarb SC, and Comb-S 2 and Comb-S 3 also show excellent compatibility with SP-SC29.

**Figure 3:**
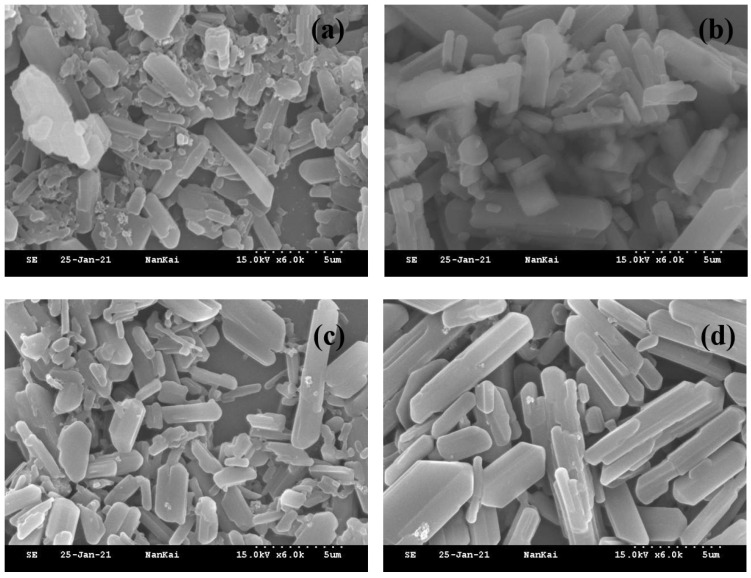
Morphology of 22% iprodione SC particles under a scanning electron microscope (SEM). (**a**) 22% iprodione SC Sample 2# (0 °C); (**b**) 22% indoxacarb SC Sample 2# (54 °C); (**c**) 22% iprodione SC Sample 5# (0 °C); (**d**) 22% indoxacarb SC Sample 5# (54 °C).

As shown in Table 3, 22% iprodione SC 2#, 4#, and 5# had particle sizes of 4.248, 4.983, and 5.194 μm, respectively, under 0 °C conditions. Under 54 °C storage conditions, the particle sizes of the SC increased to varying degrees (Figure 3). Among these samples, under all three storage conditions, 22% iprodione SC 5# exhibited the most stable particle size.

Combined with the data in Table 3, all tested dispersants, whether the commercial dispersants (SP-SC29, YUS-FS3000) or Comb-S 2, demonstrated excellent dispersing effects on 22% iprodione SC. Both of them can meet the standards for commercial application. Additionally, Comb-S 2 showed good compatibility with the commercial dispersants.

### 2.3. Other Physicochemical Parameters

In Table 4 and Table 5, the pH value of 15% indoxacarb SC ranged from 5.25 to 5.78, and its persistent foaming volume was ≤10 mL. For 22% iprodione SC, pH values fell between 5.73 and 6.60, with a persistent foaming volume also ≤20 mL. Under 54 °C, 0 °C storage conditions, no significant changes were observed in either the appearance or physicochemical properties of the indoxacarb and iprodione SC, and their stability was qualified.

In conclusion, the physicochemical properties of 15% indoxacarb SC and 22% iprodione SC can meet the application requirements. This further indicates that our dispersants can reach the application standards of commercial dispersants and exhibit good compatibility with commercial dispersants, such as SP-SC29.

### 2.4. Rheological Analysis

#### 2.4.1. Rheological Analysis of 15% Indoxacarb SC

**Figure 4:**
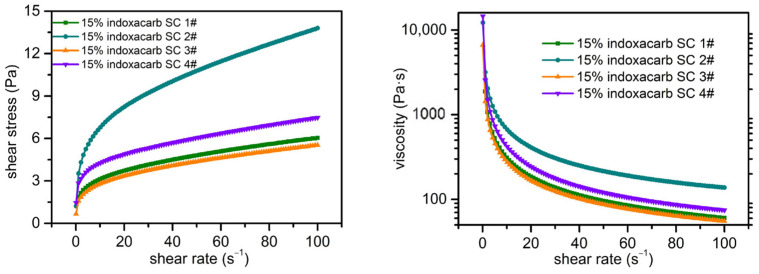
Rheological curves of 15% indoxacarb SC samples 1# to 4#.

Taking 15% indoxacarb SC 3# as an example, the rheological analysis of 15% indoxacarb SC was conducted. At shear rates below 2 s^−1^, the shear force is low. Particles in the system intertwine with each other and thus aggregate, resulting in relatively high viscosity. Among the samples, the initial viscosity of 15% indoxacarb SC 3# was 6683.5 Pa·s, which was significantly lower than that of the other three samples (Figure 4). The number of entanglements between particles decreases with increasing the shear rate. And then, the mutual entanglement between particles is disrupted. Consequently, the particles are broken down into smaller units. This causes the structure of the internal units to change under the influence of external force. Their orientation shifts to align with the direction of the shear force. Those could reduce the viscosity of the suspension system.

The fitting correlation coefficient (R^2^) values were all above 0.9941, demonstrating that the Herschel–Bulkley model is quite appropriate for the characterization of our SC (Table 6). The flow behavior index c was all less than 1, which shows that the prepared 15% indoxacarb SC exhibits the “shear-thinning” pseudoplastic characteristic. Both of those show that 15% indoxacarb SC is an ideal suspension concentrate fluid.

#### 2.4.2. Rheological Analysis of 22% Iprodione SC

**Figure 5:**
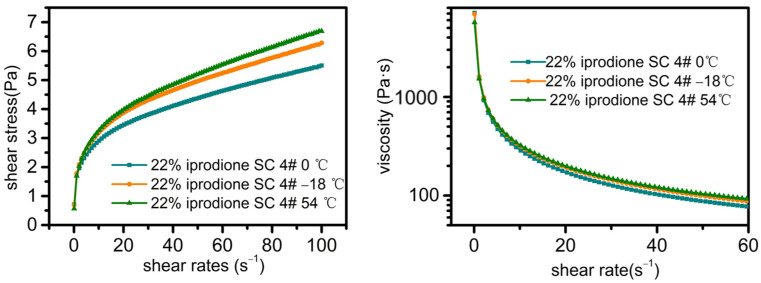
Rheological curves of 22% iprodione SC sample 4# under 0 °C, −18 °C, and 54 °C storage conditions.

For 22% iprodione SC, with the example of 4#, a rheological analysis was conducted under different storage conditions (Figure 5). At shear rates below 1.5 s^−1^, the shear stress is low, and particles in the system entangle with each other, leading to aggregation and thus high viscosity (7104.9 Pa·s). When increasing the shear rate, the number of entanglement points between particles decreases, and the mutual entanglement among particles is disrupted. Consequently, the particles break down into smaller units. This causes the structure of the internal units to change under external force. The direction will be adjusted to be consistent with the direction of the shear stress, thereby reducing the viscosity of the suspension system.

Under the three storage conditions, the rheological curves of 22% iprodione SC (4#) basically overlapped, which further confirms its excellent dispersibility and stability (Table 7).

The fitting correlation coefficient (R^2^) values are all above 0.9970, indicating that the Herschel–Bulkley model is suitable for characterizing 22% iprodione SC. The value of c suggests the “shear thinning” pseudoplastic characteristics of 22% iprodione SC. This fluid belongs to the ideal fluid for suspensions.

### 2.5. Biological Activity 

#### 2.5.1. Insecticidal Activity of 15% Indoxacarb SC

The EC_50_ values of 15% indoxacarb SC 1#, 2#, 3#, and 4# against *Spodoptera exigua* larvae were 4.5523, 3.7569, 3.3637, and 3.1734 µg·mL^−1^, respectively (Table 8). The EC_50_ value of sample 1# was significantly higher than those of the other three. This indicates that the insecticidal activity of SC prepared with either Comb-S 3 + SP-SC29, Comb-S 2 + SP-SC29, or Comb-S 1 alone is superior to that of the formulation with SP-27001 + SP-SC29. Among them, the formulation with the combined use of Comb-S 3 + SP-SC29 exhibited the best insecticidal activity.

#### 2.5.2. Fungicidal Activities of 22% Iprodione SC

The EC_50_ values of 22% iprodione SC 1#, 2#, 3#, 4#, and 5# against *Botrytis cinerea* were 1.6476, 1.5048, 1.6787, 1.7891, and 1.9413 µg·mL^−1^, respectively (Table 9). The EC_50_ values of the five samples showed little difference, with sample 2# exhibiting the best fungicidal activity. This indicates that Comb-S 2, as a dispersant, can not only replace compound commercial dispersants but also produce 22% iprodione SC with more excellent biological activity.

## 3. Materials and Methods

### 3.1. Materials

Indoxacarb TC (97%), Shandong Jingbo Agrochemical Technology Co., Ltd. (Binzhou, China); iprodione TC (95%), Jiangxi Heyi Chemical Co., Ltd. (Jiujiang, China); YUS-FS3000, Shanghai Jieshi Chemical Co., Ltd. (Shanghai, China); SP-SC29 and SP-27001, Jiangsu Qingyu Chem Chemical Technology Co., Ltd. (Yizheng, China); 1% xanthan gum, Inner Mongolia Erdos Chemical Industry Co., Ltd. (Erdos, China); magnesium aluminum silicate, Suzhou Sinoma Mineral Materials Co., Ltd. (Suzhou, China); Antifoamer1501 and S-30, Guangzhou Fangzhong Chemical Co., Ltd. (Guangzhou, China); white carbon black and ethylene glycol, Bide Pharmatech Ltd. (Shanghai, China); citric acid, Shanghai Macklin Biochemical Co., Ltd. (Shanghai, China).

### 3.2. Instruments

ME20A Electronic Balance (Mettler-Toledo Instruments Shanghai Co., Ltd., Shanghai, China); SYP Intelligent Glass Thermostatic Water Bath (Gongyi Yuhua Instrument Co., Ltd., Gongyi, China); DS-0506 Low-Temperature Thermostatic Bath (Shanghai Dusi Instrument Co., Ltd., Shanghai, China); Water-Jacketed Electric Thermostatic Incubator for Microbial Culture (Shanghai Peiyin Laboratory Instrument Co., Ltd., Shanghai, China); UPR11-5T Type Ultrapure Water Purifier (Sichuan Youpu Ultrapure Technology Co., Ltd., Chengdu, China); EQ-250DE Type Numerical Control Ultrasonic Cleaner; Laboratory Sand Mill (Jiangyin Zhuoying Drying Engineering & Technology Co., Ltd., Jiangyin, China); PHS-3C pH Meter (Shanghai Leici, Shanghai, China); BT-1600 Image Particle Analysis System (Dandong Bettersize Instrument Co., Ltd., Dandong, China); BT-9300ST Type Laser Particle Size Analyzer (Dandong Bettersize Instrument Co., Ltd., Dandong, China); S-3500N Scanning Electron Microscope (SEM) (Hitachi, Ltd., Tokyo, Japan); Agilent Technologies 1260 Infinity High-Performance Liquid Chromatograph (Agilent Technologies, Santa Clara, CA, USA); MCR-102 Anton Paar Rheometer (Anton Paar, Graz, Austria).

### 3.3. Preparation of 15% Indoxacarb SC and 22% Iprodione SC

SP-SC29 is a polymeric anionic-nonionic surfactant with a structure similar to “Gemini”. The product can be used in SC alone or in combination with a polymeric dispersant. SP-27001 is a polycarboxylate-type dispersant that can be applied to high-concentration aqueous suspension systems. It has good adaptability and is often used in combination with SP-SC29 for application in pesticide suspensions. YUS-FS3000 is a phosphate-type anionic dispersing emulsifier, widely used in dispersant formulations. We prepared a non-ionic dispersant with a structure similar to polycarboxylates. To study its compatibility with SP-SC29, SP-27001, and YUS-FS3000, and also its effect when used alone, we designed the formulations in Table 10 and Table 11.

Water, ethylene glycol, different types of dispersants, and indoxacarb or iprodione were added to the beaker in sequence and stirred evenly. The white carbon black, xanthan gum, magnesium aluminum silicate, 1501, and citric acid (addition amount according to the dosage form formula in Table 10 and Table 11) were added. After stirring evenly, it was added to the sand mill, and finally, 140 g of zirconium silicate beads (with a diameter of 0.8 mm) was added. The mixture was ground at room temperature for 2 h at a rotational speed of 1440 rpm. After grinding, the zirconium beads were filtered out to obtain indoxacarb or iprodione suspension concentrate (SC).

### 3.4. Content Determination

#### 3.4.1. Sample Preparation

An accurately weighed 20 mg sample of indoxacarb technical material (99%) or iprodione (97.6%) (accurate to 0.0001 g) was dissolved in acetonitrile and diluted to a final volume of 50 mL in a volumetric flask to obtain a standard solution.

For the preparation of indoxacarb SC or iprodione SC, 0.12 g of each SC was accurately weighed and diluted to the mark with acetonitrile in a 50 mL volumetric flask.

#### 3.4.2. HPLC Operating Conditions

For 15% Indoxacarb SC

Column model: Agilent Poroshell 12 EC; mobile phase: volume ratio ψ (acetonitrile/water) = 75:25 (filtered through a membrane filter and degassed); flow rate: 1.0 mL/min; column temperature: 30 °C; detection wavelength: 280 nm; injection volume: 5 μL.

For 22% Iprodione SC

Column model: Agilent Poroshell 12 EC; mobile phase: volume ratio ψ (acetonitrile/water/glacial acetic acid) = 60:40:0.5 (filtered through a membrane filter and degassed); flow rate: 1.0 mL/min; column temperature: 25 °C; detection wavelength: 220 nm; injection volume: 5 μL.

The mass concentration of the sample is calculated according to the following formula:

$$w_{2}=\frac{A_{2}m_{1}w_{1}}{A_{1}m_{2}}$$

*w*_1_: refers to the mass fraction of the active ingredient in the standard sample solution (%); *w*_2_: refers to the mass fraction of the active ingredient in the sample solution (%); *A*_1_: refers to the peak area of the standard sample solution; *A*_2_: refers to the peak area of the sample solution; *m*_1_: refers to the mass of the standard sample (g); *m*_2_: refers to the mass of the sample (g).

### 3.5. Suspensibility Determination

The determination of the suspension rate of suspension concentrate (SC) refers to the national standard (GB/T 14825-2006) [28].

The suspension rate of the active ingredient in the sample, denoted as *w*_1_(%), is calculated according to the following formula:

$$w_{1}=\frac{m_{1}-m_{2}}{m_{1}}\times \frac{10}{9}\times 100$$

In the formula, *m*_1_: mass of the active ingredient in the sample taken for preparing the suspension (g); *m*_2_: mass of the active ingredient in the 25 mL suspension at the bottom of the graduated cylinder (g); 10/9: conversion factor.

### 3.6. Particle Size Determination

Slowly add the pesticide aqueous suspension concentrate (SC) dropwise into the sample measurement cell of the laser particle size analyzer while stirring. After complete dispersion, pass a laser beam through the sample cell, and use the BT-9300ST Laser Particle Size Analyzer (Dandong, China) to test different suspension concentrates.

### 3.7. Morphology Characterization

#### 3.7.1. Optical Microscope

Dilute 0.1 g of the pesticide aqueous suspension concentrate (SC) to 20 mL with deionized water. After stirring until well-mixed, take one drop and place it on a glass slide. Use the BT-1600 Image Particle Analysis System (Dandong, China) to observe its microstructure under a microscope at 400× magnification.

#### 3.7.2. Scanning Electron Microscope

Drop the 200-fold diluted pesticide aqueous suspension concentrate (SC) onto a silicon wafer. After drying, sputter-coat the sample with gold. Test it using the S-3500N Scanning Electron Microscope (Tokyo, Japan) at 15 kV to investigate the aggregation state of micro-particles in the pesticide aqueous suspension concentrate under different storage temperatures.

### 3.8. pH Value Test

Determine its pH value with reference to the GB/T 1601-93 [29].

### 3.9. Foam Persistence Test

Determine its persistent foaming property with reference to the GB/T 28137-2011 [30].

### 3.10. Stability Test

The stability tests include two standard scenarios: thermal storage (54 °C) and low-temperature (0 °C). Additionally, due to the large day–night temperature differences in northern China during winter, where pesticides may experience intermittent freezing and thawing, we added a more stringent test condition: stability testing under freezing (−18 °C) environment.

Specifically, the thermal storage stability was determined with reference to the GB/T 19136-2003 [31]; the low-temperature stability was determined with reference to the GB/T 19137-2003 [32].

### 3.11. Rheological Test

The variation patterns of viscosity and shear stress of the suspension concentrate (SC) were studied with shear rates ranging from 0.1 to 100 s^−1^ at 25 °C (tested with a MCR-102 Anton Paar rheometer, rotor model: PP17; temperature set at 25 °C).

Further, fit the rheological data of the suspension concentrate using the Herschel–Bulkley model. Analyze the rheological stability and fluid type of the pesticide aqueous suspension concentrate by comparing the model’s yield value (*τ*_0_) and the magnitude of the correlation coefficient (R^2^). The Herschel–Bulkley model is as follows:

$$\tau =\tau_{0}+k\gamma^{c}$$

In the formula, *τ*: shear stress (unit: Pa); *γ*: shear rate (unit: s^−1^); *τ*_0_: yield stress (unit: Pa), also referred to as the yield value; *k*: consistency index (unit: Pa·s^n^).

### 3.12. Biological Activity Study

#### 3.12.1. Bioactivity Test of 15% Indoxacarb SC

The cabbage leaf disk spray method was adopted to conduct the test, with *Spodoptera exigua* (beet armyworm) larvae as subjects.

#### 3.12.2. The Fungicidal Activity of 22% Iprodione SC

*Botrytis cinerea* (cucumber gray mold pathogen) was selected as the subject. The experiment was as follows: inoculate the pathogens onto Potato Dextrose Agar (PDA) medium and incubate for 7 days. A 4 cm diameter mycelial disk was prepared on the edge of the colony with a hole puncher and inoculated on PDA medium containing 50 μg mL^−1^ and no agent. The colony diameter was measured.

## 4. Conclusions

In this study, novel comb-type fluorinated polymeric surfactants (Comb-S 1, Comb-S 2, Comb-S 3) and commercial dispersants (SP-27001, SP-SC29, and YUS-FS3000) were used as dispersing agents to prepare 15% indoxacarb SC and 22% iprodione SC. The physical and chemical properties, as well as indoor biological activities (insecticidal and fungicidal activities) of the two formulations were determined. The results showed that for 15% indoxacarb SC, the formulations prepared with either compounded commercial dispersants, Comb-S 2/Comb-S 3 compounded with SP-SC29, or Comb-S 1 all exhibited good dispersibility, stability, and comprehensive application effects. Moreover, the effect of Comb-S 1 used alone was comparable to that of compounded adjuvant systems, showing potential to replace compounded adjuvants. The results show that the insecticidal activity of SCs prepared by Comb-S 3 + SP-SC29, Comb-S 2 + SP-SC29, and Comb-S 1 is better than that of SP-27001 + SP-SC29, and the best compound is Comb-S 3 + SP-SC29. For 22% iprodione SC, both the system (SP-SC29 + YUS-FS3000 and SP-SC29+ Comb-S 2) and Comb-S 2 alone could endow the formulations with good dispersion effects, and meet the standards for commercial application. In addition, Comb-S 2 exhibited good compatibility with commercial dispersants, while its use alone also exhibited excellent dispersing properties. The fungicidal activity showed that Comb-S 2, as the dispersant, not only replaces compounded commercial dispersants, but the prepared 22% iprodione SC has better biological activity. In conclusion, the novel comb-type fluorinated polymeric surfactants show good application potential in water-based pesticide formulations and deserve further in-depth research.

## Figures and Tables

**Table 1:** Composition and GPC data of of surfactants.

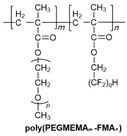	**poly(PEGMEMA*_m_*-FMA*_n_*)**	***p***	***q***	***m*/*n* ^1^**	***M_n_^2^ × 10^4^ (Daltons)***	***M_w_^3^ × 10^4^ (Daltons)***	**PDI ^4^**
	Comb-S ^1^	4	2	3/1	1.644	6.764	4.11
	Comb-S ^2^	4	6	3/1	2.067	6.197	3.00
	Comb-S ^3^	20	6	3/1	1.745	2.191	1.26

^1^ Molar feed ratio of PEGMEMA/FMA; ^2^ number average molecular weight; ^3^ mass average molar mass; ^4^ polydispersity.

**Table 2:** Physicochemical parameters (1) of 15% indoxacarb SC.

Formulation Number	Dispersant	Storage Temperature/°C	Nominal Size (μm)			Active Ingredient Content/%	Suspensibility/%
			D10	D50	D90		
15% indoxacarb SC 1#	SP-27001 SP-SC29	54	0.850	2.433	6.898	15.35	97.29
		0	0.640	1.804	4.964	15.19	98.57
		−18	0.595	1.769	4.870	15.43	98.78
15% indoxacarb SC 2#	Comb-S 1	54	0.894	3.998	10.69	16.08	95.22
		0	0.602	1.497	3.520	14.70	99.53
		−18	0.610	1.554	3.695	15.12	97.15
15% indoxacarb SC 3#	Comb-S 2 SP-SC29	54	0.867	2.481	6.832	15.82	98.08
		0	0.558	1.591	4.208	15.36	98.95
		−18	0.556	1.607	4.419	15.21	98.39
15% indoxacarb SC 4#	Comb-S 3 SP-SC29	54	0.753	2.058	5.483	15.14	97.07
		0	0.562	1.648	4.550	14.37	98.93
		−18	0.632	2.314	5.885	14.06	96.53

**Table 3:** Physicochemical parameters (1) of 22% iprodione SC.

Formulation Number	Dispersant	Storage Temperature/°C	Nominal Size (μm)			Active Ingredient Content/%	Suspensibility/%
			D10	D50	D90		
22% iprodione SC 1#	SP-SC29 SP-27001	54	1.099	3.261	8.742	23.06	95.87
		0	0.818	2.344	5.556	23.04	98.64
		−18	0.910	3.190	9.193	22.74	56.48
22% iprodione SC 2#	Comb-S 2	54	1.064	4.421	12.01	23.62	91.61
		0	0.708	1.884	4.248	23.31	99.08
		−18	0.775	2.469	6.139	23.20	96.28
22% iprodione SC 3#	Comb-S 2 SP-27001	54	1.080	3.202	8.806	23.30	96.32
		0	0.795	2.199	5.013	23.18	99.11
		−18	0.872	3.061	9.395	22.60	65.36
22% iprodione SC 4#	SP-SC29 Comb-S 2	54	1.085	3.439	9.155	22.91	93.52
		0	0.775	2.152	4.983	23.11	99.08
		−18	0.815	2.409	5.663	23.03	96.10
22% iprodione SC 5#	SP-SC29 YUS-FS3000	54	1.026	3.041	8.378	23.55	96.18
		0	0.788	2.207	5.194	23.09	97.31
		−18	0.771	2.195	5.171	22.91	95.76

**Table 4:** Physicochemical parameters (2) of 15% indoxacarb SC.

Physicochemical Parameter	15% Indoxacarb SC 1#	15% Indoxacarb SC 2#	15% Indoxacarb SC 3#	15% Indoxacarb SC 4#
pH value	5.43	5.74	5.25	5.78
Persistent foam (mL)	6	0	8	10
Dispersity	Good	Good	Good	Good
Stability at 54 °C	Qualified	Qualified	Qualified	Qualified
Stability at 0 °C	Qualified	Qualified	Qualified	Qualified

**Table 5:** Physicochemical parameters (2) of 22% iprodione SC.

Physicochemical Parameter	22% Iprodione SC 1#	22% Iprodione SC 2#	22% Iprodione SC 3#	22% Iprodione SC 4#	22% Iprodione SC 5#
pH value	6.53	6.60	6.58	5.73	5.79
Persistent foam (mL)	4	18	20	8	10
Dispersity	Good	Good	Good	Good	Good
Stability at 54 °C	Qualified	Qualified	Qualified	Qualified	Qualified
Stability at 0 °C	Qualified	Qualified	Qualified	Qualified	Qualified

**Table 6:** Rheological parameters of 15% indoxacarb SC.

Formulation Number	$\tau_{0}$	k	c	R^2^
15% indoxacarb SC 1#	1.2402	0.7183	0.4112	0.9996
15% indoxacarb SC 2#	0.5110	2.8485	0.3307	0.9977
15% indoxacarb SC 3#	0.4443	1.0454	0.3405	0.9980
15% indoxacarb SC 4#	1.3623	1.3039	0.3310	0.9941

$\tau_{0}$: yield stress (unit: Pa, pascal), k: consistency index (unit: Pa·sn), c: flow behavior index of the fluid.

**Table 7:** Rheological parameters of 22% iprodione SC.

22% Iprodione SC 4#	$\tau_{0}$	k	c	R^2^
0 °C	0.3949	1.1932	0.3115	0.9970
−18 °C	0.2253	1.4321	0.3087	0.9980
54 °C	0.2371	1.3606	0.3346	0.9975

$\tau_{0}$: yield stress (unit: Pa), k: consistency index (unit: Pa·s^n^), c: flow behavior index of the fluid.

**Table 8:** Insecticidal activity of 15% indoxacarb SC against *Spodoptera exigua* larvae.

Formulation Number	y = ax + b	EC_50_ (µg mL^−1^)
15% indoxacarb SC 1#	y = 3.8473x + 2.4675	4.5523
15% indoxacarb SC 2#	y = 3.8976x + 2.7596	3.7569
15% indoxacarb SC 3#	y = 2.8705x + 3.4878	3.3637
15% indoxacarb SC 4#	y = 2.5169x + 3.7377	3.1734

**Table 9:** Fungicidal activities of 22% iprodione SC.

Formulation Number	y = ax + b	EC_50_ (µg mL^−1^)
22% iprodione SC 1#	y = 0.6267x + 4.8641	1.6476
22% iprodione SC 2#	y = 1.2913x + 4.7708	1.5048
22% iprodione SC 3#	y = 0.8570x + 4.8072	1.6787
22% iprodione SC 4#	y = 1.0058x + 4.7459	1.7891
22% iprodione SC 5#	y = 0.9371x + 4.7300	1.9413

**Table 10:** Formulation of 15% indoxacarb SC.

Ingredient	Formula (g)	15% Indoxacarb SC1#	15% Indoxacarb SC2#	15% Indoxacarb SC3#	15% Indoxacarb SC4#
Technical material (15%)	15.5	97% Indoxacarb TC			
Dispersant (3%)	3	SP-27001	Comb-S 1	Comb-S 2	Comb-S 3
Dispersant (2%)	2	SP-SC29		SP-SC29	SP-SC29
Thickener (1%)	1	Magnesium aluminum silicate			
Thickener (0.2%)	0.2	1% Xanthan gum			
Preservatives (0.2%)	0.2	S-30			
Anticaking agent (1%)	1	White carbon black			
Antifreeze (4%)	4	Ethylene glycol			
pH regulator (0.2%)	0.2	Citric acid			
Antifoamer (0.2%)	0.2	1501			
Solvent	72.7	Deionized water			

**Table 11:** Formulation of 22% iprodione SC.

Ingredient	Formula (g)	22% Iprodione SC 1#	22% Iprodione SC 2#	22% Iprodione SC 3#	22% Iprodione SC 4#	22% Iprodione SC 5#
Technical material (22%)	32.5	95% Iprodione TC				
Dispersant (2%)	2.8	SP- SC29	Comb-S 2	Comb-S 2	SP-SC29	SP-SC29
Dispersant (3%)	4.2	SP-27001		SP-27001	Comb-S 2	YUS-FS3000
Thickener (0.5%)	0.7	Magnesium aluminum silicate				
Thickener (0.2%)	0.28	1% Xanthan gum				
Preservatives (0.2%)	0.28	S-30				
Anticaking agent (1%)	1.4	White carbon black				
Antifreeze (4%)	5.6	Ethylene glycol				
Antifoamer (0.2%)	0.3	1501				
Solvent	91.94	Deionized water				

## Data Availability

The original contributions presented in this study are included in the article. Further inquiries can be directed to the corresponding authors.
